# Evolution within a language: environmental differences contribute to divergence of dialect groups

**DOI:** 10.1186/s12862-018-1238-6

**Published:** 2018-09-03

**Authors:** Terhi Honkola, Kalle Ruokolainen, Kaj J. J. Syrjänen, Unni-Päivä Leino, Ilpo Tammi, Niklas Wahlberg, Outi Vesakoski

**Affiliations:** 1 Department of Biology, FI-20014 University of Turku, Turku, Finland; 2 Institute of Estonian and General Linguistics, Jakobi 2, University of Tartu, 51014 Tartu, Estonia; 3 Department of Geography and Geology, FI-20014 University of Turku, Turku, Finland; 4 Faculty of Communication Sciences, FI-33014 University of Tampere, Tampere, Finland; 5 Council of Tampere Region, FI-33201 Tampere, Tampere, Finland; 6 Department of Biology, Sölvegatan 37, Lund University, 223 62 Lund, Sweden

**Keywords:** Dialect divergence, Linguistic microevolution, Multiple regression on distance matrices, Variation partitioning, Human ecology, Environmental variation, Cultural adaptation, Linguistic diversity

## Abstract

**Background:**

The processes leading to the diversity of over 7000 present-day languages have been the subject of scholarly interest for centuries. Several factors have been suggested to contribute to the spatial segregation of speaker populations and the subsequent linguistic divergence. However, their formal testing and the quantification of their relative roles is still missing. We focussed here on the early stages of the linguistic divergence process, that is, the divergence of dialects, with a special focus on the ecological settings of the speaker populations. We adopted conceptual and statistical approaches from biological microevolution and parallelled intra-lingual variation with genetic variation within a species. We modelled the roles of geographical distance, differences in environmental and cultural conditions and in administrative history on linguistic divergence at two different levels: between municipal dialects (cf. in biology, between individuals) and between dialect groups (cf. in biology, between populations).

**Results:**

We found that geographical distance and administrative history were important in separating municipal dialects. However, environmental and cultural differences contributed markedly to the divergence of dialect groups. In biology, increase in genetic differences between populations together with environmental differences may suggest genetic differentiation of populations through adaptation to the local environment. However, our interpretation of this result is not that language itself adapts to the environment. Instead, it is based on *Homo sapiens* being affected by its environment, and its capability to adapt culturally to various environmental conditions. The differences in cultural adaptations arising from environmental heterogeneity could have acted as nonphysical barriers and limited the contacts and communication between groups. As a result, linguistic differentiation may emerge over time in those speaker populations which are, at least partially, separated.

**Conclusions:**

Given that the dialects of isolated speaker populations may eventually evolve into different languages, our result suggests that cultural adaptation to local environment and the associated isolation of speaker populations have contributed to the emergence of the global patterns of linguistic diversity.

**Electronic supplementary material:**

The online version of this article (10.1186/s12862-018-1238-6) contains supplementary material, which is available to authorized users.

## Background

More than 7000 languages exist in the world today [1]. Many of these languages have emerged through a process of linguistic divergence, in which one linguistic unit separates over time into more or less distinct units. To comprehend the emergence of diversity, an understanding of the mechanisms of the divergence process is required. In biology, mechanisms of species divergence can be studied by focussing on population divergence within a framework of microevolution [2, 3]. Here, we adopted certain elements of a biological microevolutionary approach in studying the initial stages of linguistic divergence, i.e. the divergence of dialects.

Adopting elements from biological approaches to study language-related questions is possible due to the roughly analogous nature of species and languages [4, 5]. In both cases individuals carry heritable material (genetic material or linguistic information), which comprises various features (cf. loci in genetics) and variants therein (cf. alleles in genetics). As individuals may differ from each other in the variants they possess, they may be grouped together into populations and dialect groups based on the similarity of their variants (for more detailed discussion see [5]).

Linguistic divergence has been described as a process of “inter-group boundary formation”, [6] referring to the linguistic differentiation of human populations due to constrained communication between them. Generally recognised factors restricting communication between human populations include environmental and geographical barriers (e.g. dense forests, swamps, mountains or oceans) [7–9] and geographical distance [7, 10–13]. The influence of these factors on the intensity of communication are further shaped by human-related factors such as social, cultural and political settings [7, 12, 13]. Barriers and geographical distance isolate speaker populations by acting as physical hindrances to movement, while human-related factors may also isolate populations via social group cohesion by encouraging communication within the groups and discouraging it between them.

These different isolating factors are not mutually exclusive [7, 12–14]. Nevertheless, their relative roles have not, to our knowledge, been attested quantitatively. Much of the work on linguistic divergence has been non-quantitative, and in the existing quantitative studies, only one factor at a time has commonly been studied [11]. Furthermore, it has not been quantitatively studied whether mere differences in environmental conditions may induce separation between speaker populations and thus, their language. This is, nevertheless, a plausible hypothesis, as environmental differences separate the populations of other species [15] and similar to these species, *Homo sapiens* is dependent on its environment.

In this study, we addressed and combined the two aspects that are fundamental in understanding the emergence of global linguistic diversity: linguistic divergence in the context of the ecological settings of the speaker populations and its relative role to other contributing factors. We achieved this by parallelling genetic variation clustered within populations with linguistic variation clustered within dialect groups. In practice, we studied whether linguistic divergence and the related isolation of speaker populations is explained by differences in ecological environment, as is done in evolutionary ecology when studying ecological speciation [16–18], or whether linguistic divergence is explained by differences in culture, geographical distance, and/or differences in administrative history.

In biology, the idea of population divergence via adaptation to differing ecological environments was brought up in the 1940s [19, 20], but studies on ecological speciation as a mechanism for population divergence have gained momentum only recently [18, 21]. In ecological speciation, the genetic differentiation of populations occurs through local adaptation in differing selective environments, even if there is ongoing gene flow [22]. Adaptation takes place in certain loci, but genetic differentiation via adaptation may also be detected indirectly in selectively neutral genetic markers, such as microsatellites [23, 24]. This is possible as neutral parts of the genome may differentiate due to genetic linkage [25]. Differentiation without genetic linkage is also possible if a divergent selection of multiple loci is strong enough to reduce the average rate of effective migration between environments [26]. Thus, sufficiently strong environmental differences may limit successful gene flow from one environment to another, eventually developing genetic differentiation between populations [24, 27, 28]. Accordingly, when gene exchange is stronger between similar environments and weaker when they differ, a pattern emerges where genetic differences between populations increase with environmental differences [23, 24]. This pattern can be called isolation by environment (IBE). Notably, IBE indicates that adaptive processes have contributed to the divergence process, as when IBE is detected the populations do not differentiate randomly but in relation to the differences in their biotic surroundings [23, 24, 29].

We hypothesise linguistic IBE would refer, correspondingly, to a situation where linguistic differences between speaker populations increase together with differences in their environmental conditions. Linguistic IBE could be interpreted as an outcome of reduced communication between speaker populations living in different environmental conditions. Following the biological inference, this would indicate that the disruption of contacts between speaker populations is due to environmental differences and adaptations related to these environments.

The next question then is, what type of adaptive process may be detected from linguistic IBE? Firstly, linguistic adaptations do not in general have effects on the biological fitness of language speakers, as “forms of words themselves do not have fitness implications” [30]. However, language is considered to be a part of human culture, and as it lacks the fitness effects, it may be seen as a neutral marker of cultural history of human populations [30]. Secondly, it is known that humans may adapt to their environment both genetically [31, 32] and culturally [33]. For example, subsistence strategies are cultural features which have a direct connection to the biological fitness of an individual [33]. Due to the connection between language and culture and the possible fitness implications of certain cultural features, we conclude that the adaptive process inferable from linguistic IBE is cultural adaptation and not linguistic adaptation. In other words, we are not studying whether language adapts to environment, but instead whether language speakers adapt to their local environment via their culture.

While language may be transmitted together with other cultural features, these may also be passed on separately from each other [34]. Therefore, our investigation also explored the strength of the relationship between linguistic and cultural differentiation among populations by measuring the correlation between linguistic and cultural differences. We refer to the pattern where linguistic differences increase together with cultural differences as isolation by culture (IBC).

Geographical distance can contribute to the separation of biological populations by limiting dispersal and gene flow, thus setting the stage for genetic drift within populations [23]. Genetic drift, or random changes in the allele frequencies of populations, represents the neutral process of evolution. In the absence of geographical barriers and selection pressures inducing divergence, neighbouring populations should share more gene flow than distant populations. In this scenario, the resulting pattern is a gradual increase in genetic differences as a function of increasing geographical distance [35], called isolation by distance (IBD). If IBD is observed independently of environmental differences, it is seen as a signal that neutral processes – rather than adaptation – are the major forces structuring the spatial pattern of genetic variation [29]. Correspondingly, linguistic IBD refers to a pattern where linguistic differences increase together with geographical distance [36].

Administrative histories have influenced the cultural and social interactions of humans, and thus their languages, throughout history [37, 38]. Their isolating effect may work in two ways: administrative borders may physically prevent contact, and social cohesion within administrative borders, for example due to kinship relationships and religious beliefs, may keep groups separated [39]. Here, we studied the role of administrative history in separating language speakers and their linguistic variants from each other. In practice, we studied whether differences in administrative history coincide with linguistic differences, a pattern we term “isolation by administrative history” (IBA).

Within the adopted population genetic framework, we can model the relative roles of different factors explaining linguistic divergence. In addition to that, we can make inferences about the relative roles of the processes driving linguistic divergence [29]. As explained above, we first studied the spatial covariation of linguistic differences and extra-linguistic factors. Following this, we inferred from the observed patterns of IBD and IBE the relative roles of neutral (IBD) and adaptive (IBE) processes contributing to the linguistic divergence. The larger relative role of environmental differences (IBE) suggests that linguistic divergence was caused by cultural adaptation of the language speakers to their local environment. However, if dialect divergence were coupled with geographical isolation, we would observe a marked relative role of geographical distance (IBD). In this case, neutral processes would act as the main driver of linguistic differentiation.

As our study object, we used dialects of the Finnish language. The dataset, a digitised edition of the Dialect Atlas of Finnish [5, 40, 41], represents linguistic variation in the Finnish language as it existed almost 100 years ago [42]. The data were collected per municipality (local administrative unit [5]; *n* = 471 in this study) and they represent linguistic variation within Finland before urbanisation, industrialisation and mass media had evened out dialect differences [43]. The Dialect Atlas of Finnish represents the spatial variation of language and regional dialects, and not social variation and sociolects. The dataset thus provides us an opportunity to study the initial stages of spatial separation of linguistic populations with a population genetic approach. We have shown earlier that this data is compatible with population genetic analyses [5].

We studied the association of extra-linguistic factors with linguistic divergence and therefore essentially also on the isolation of human populations. Extra-linguistic factors include geographical distances among the studied units, and data on 18 environmental and 22 cultural variables representing the environmental and cultural surroundings more than a century ago (Additional file 1: Table S1). We also compiled data on administrative history, including 16 sets of administrative borders from the thirteenth to the nineteenth century. Some variables within this dataset have a wider temporal coverage than the Dialect Atlas, (e.g. variation in soil types is considered to be unchanging), and the data aims to capture the extra-linguistic settings of Finland during the last one thousand years, during which the contemporary Finnish dialects have largely taken shape [44].

**Fig. 1:**
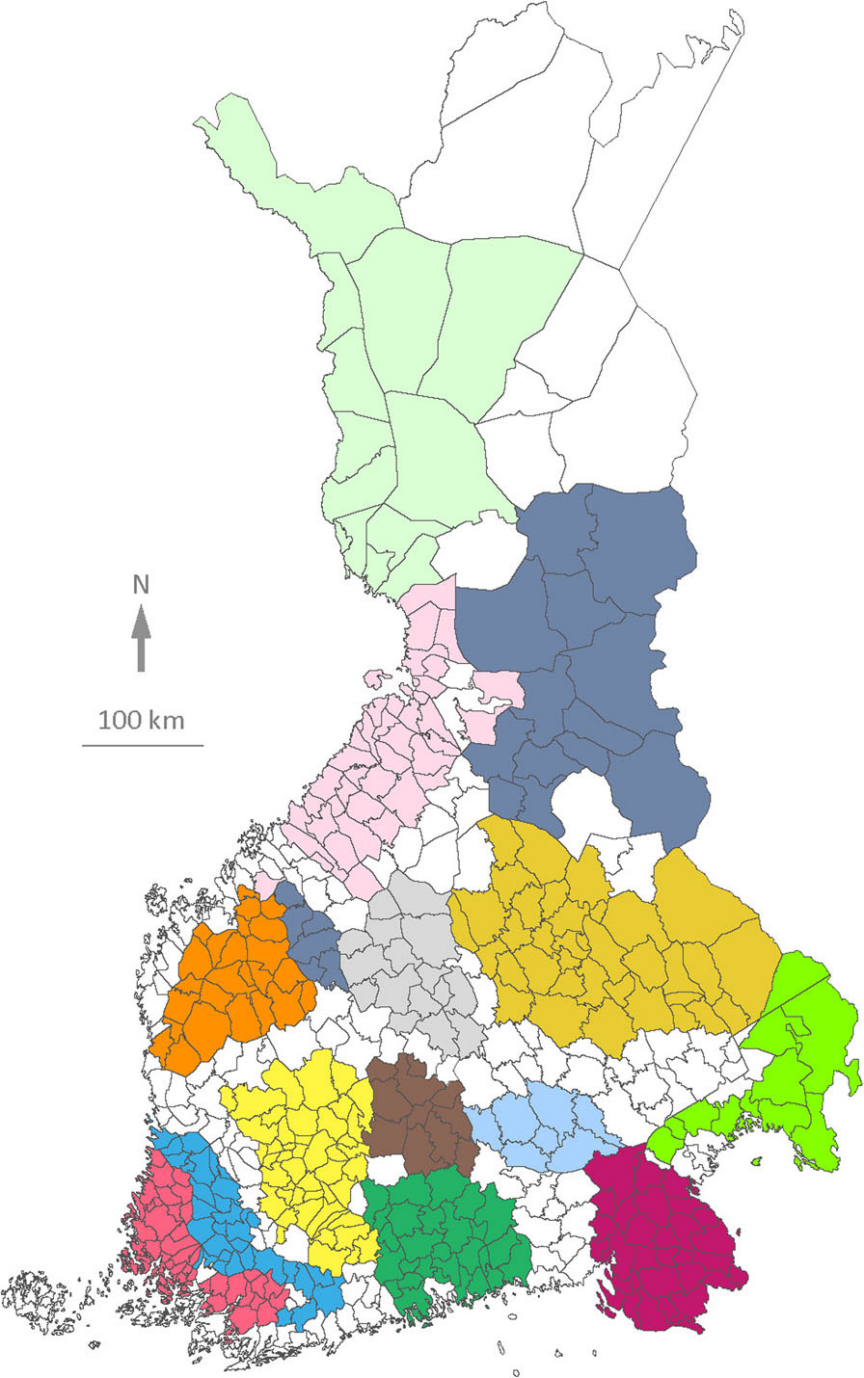
Fourteen dialect groups of the Finnish language. Clustering produced with the admixture model of the Structure software package [66], showing the core areas of the dialect groups (areas with IC values ranging 0.75–1) with different colours. National and municipal borders are shown as they were in the 1920s. Municipalities shown in white represent either transitional dialect areas (IC value below 0.75), which were not included in the dialect group analyses, or the Swedish-speaking areas in the southern and western coasts, which are not covered by the Dialect Atlas [42]

In our language data, the linguistic variants are documented per municipality instead of individual language speakers. Accordingly, in our analyses we parallelled municipalities and their municipal dialects with biological individuals. We clustered linguistic variation of municipal dialects into dialect groups in a similar way to how genetic variation of individuals would be clustered into populations. We inferred the dialect groups with a population genetic model-based clustering method (Fig. 1). We previously found that dialect groups produced by this method are largely in line with the traditional dialect divisions of Finnish [5].

We studied linguistic divergence at two spatio-linguistic resolutions: between municipal dialects (collected per municipality) and between dialect groups (clusters of municipal dialects). First, we studied the divergence of municipal dialects. We built one model where we explained linguistic differences between municipal dialects with environmental and cultural differences and with geographical distance, and another model where differences in administrative history were also included. We expected the role of geographical distance to be high due to spatial autocorrelation in linguistic variation [11]. Our expectations about the explanatory power of environmental and cultural differences, and of the differences in administrative history, were less clear. Next, we examined which factors have a role in the divergence of dialect groups. We did that with a model where we explained linguistic differences between dialect groups with environmental and cultural differences and with geographical distance. Thus, in total we had three different models: two with municipal dialects and one with dialect groups. The effect of administrative history was not studied with the dialect groups, as these typically encompass multiple administrative areas. Based on the relative roles of these factors in explaining the differences between the dialect groups, we made deductions about neutral and adaptive processes that might be responsible for inducing the divergence of dialect groups. We inferred the evolutionary processes from the analyses of the dialect groups only, as evolution is a property of a population (defined as a change of allele frequencies in a population), not of an individual (Table 1). Here we discuss in particular the role of natural environment as an isolating force, as this has been relatively unstudied compared to the isolating force of sociolinguistic processes [45].

**Table 1:** Factors and patterns studied, and the evolutionary processes inducing linguistic divergence

	Dialect groups	Municipal dialects
Studied factors		
Geographical distance	x	x
Environmental differences	x	x
Cultural differences	x	x
Difference in administrative history		x
Studied patterns		
IBD	x	x
IBE	x	x
IBC	x	x
IBA		x
Evolutionary processes		
IBD – neutral	x	
IBE – adaptive	x	

Evolutionary processes were inferred from the studied patterns. Environmental and cultural factors include several different variables (Additional file 1: Table S1). Administrative history is a compilation of 16 sets of administrative borders from different historical times

The data were analysed with multiple regression on distance matrices (MRM; [46, 47]), which allows simultaneous analysis of several explanatory variables. MRM is an extension of the Mantel test [48], where both the response and explanatory variables are dissimilarity matrices. Error probabilities of type one were estimated through permutations. Linguistic differences between the dialect groups were measured as F_ST_ values (introduced for languages in [5]). Linguistic differences between municipal dialects were calculated following Séguy’s dialect distance metric, i.e. as a percentage of disagreeing linguistic features between pairs of municipal dialects [49]. We specifically chose the response variable to be linguistic difference and not language itself. This is because we are interested why the spoken language is sometimes more and sometimes less different between localities, not why a certain kind of language variety appears in a certain locality. In addition, by defining the response variable as a distance (or difference), we were also able to include the geographical distance, an often-discussed potential dispersal barrier [7, 10–13], as an explanatory variable in the multiple regression analysis. The other explanatory variables used in our analyses were a distance matrix representing administrative history, and 22 cultural and 18 environmental variables turned into distance matrices (see Methods and Additional file 1: Table S1).

In this study, we aim to resolve the inducers of linguistic divergence in the early stages of the divergence process. In other words, we want to disentangle the relative importance of different 1) extra-linguistic factors in the divergence of municipal dialects and dialect groups and 2) evolutionary processes prompting the divergence of dialect groups.

## Results

**Fig. 2:**
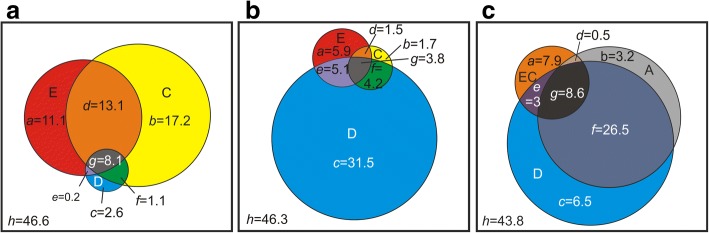
Partitioning the total variation in linguistic differences to components explained by variation in extra-linguistic factors. The extra-linguistic factors are environmental (E), cultural (C), geographical (D), joined environment-cultural (EC) and administrative (A) distances. (**a**) The relative proportions of E, C and D in explaining linguistic differences between the dialect groups. (**b**) The relative proportions of E, C and D, and (**c**) the relative proportions of A, D and EC in explaining linguistic differences between the municipal dialects. The values present the percentages of individual (*a-c*) and joint (*d-g*) contributions of explanatory factors; *h* refers to the amount of unexplained variation. Circle sizes roughly represent percentages of the total variation

The three extra-linguistic factors (environmental, cultural and geographical distances) explained somewhat overlapping parts of the variation in linguistic differences (Fig. 2a and b). In all, these factors explained over half of the linguistic variance both at the levels of dialect groups and municipal dialects (53.4% and 53.7%, respectively). However, the relative explanatory powers of the three factors differed notably between these two levels (Fig. 2a and b). Adding administrative distances to the analyses of the municipal dialects (Fig. 2c) only slightly increased the overall explanatory power of the model (56.2%). Nevertheless, this addition markedly decreased the fraction of pure geographical distance compared to the analysis of municipal dialects where administrative distances were not included (Fig. 2b).

**Fig. 3:**
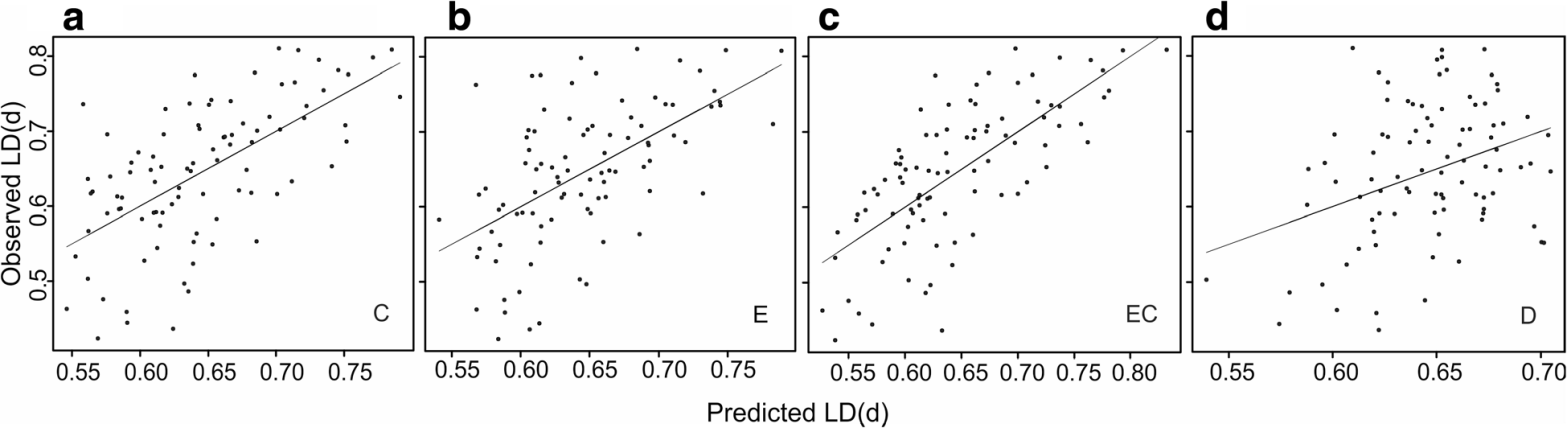
Observed vs predicted linguistic differences between the dialect groups. Predicted differences between the dialect groups calculated on the basis of (**a**) cultural differences (corresponds to fractions *b, d, f* and *g* in Fig. 2a), (**b**) environmental differences (corresponds to fractions *a, d, e*, and *g* in Fig. 2a), (**c**) environmental and cultural differences together (corresponds to fractions *a, b, d, e, f* and *g* in Fig. 2a), and (**d**) geographical distances (corresponds to fractions *c, e, f* and *g* in Fig. 2a). Cultural and environmental features are given in Table 2. *N* = 91, as pairing up the 14 dialect groups resulted with 91 linguistic difference values

**Fig. 4:**
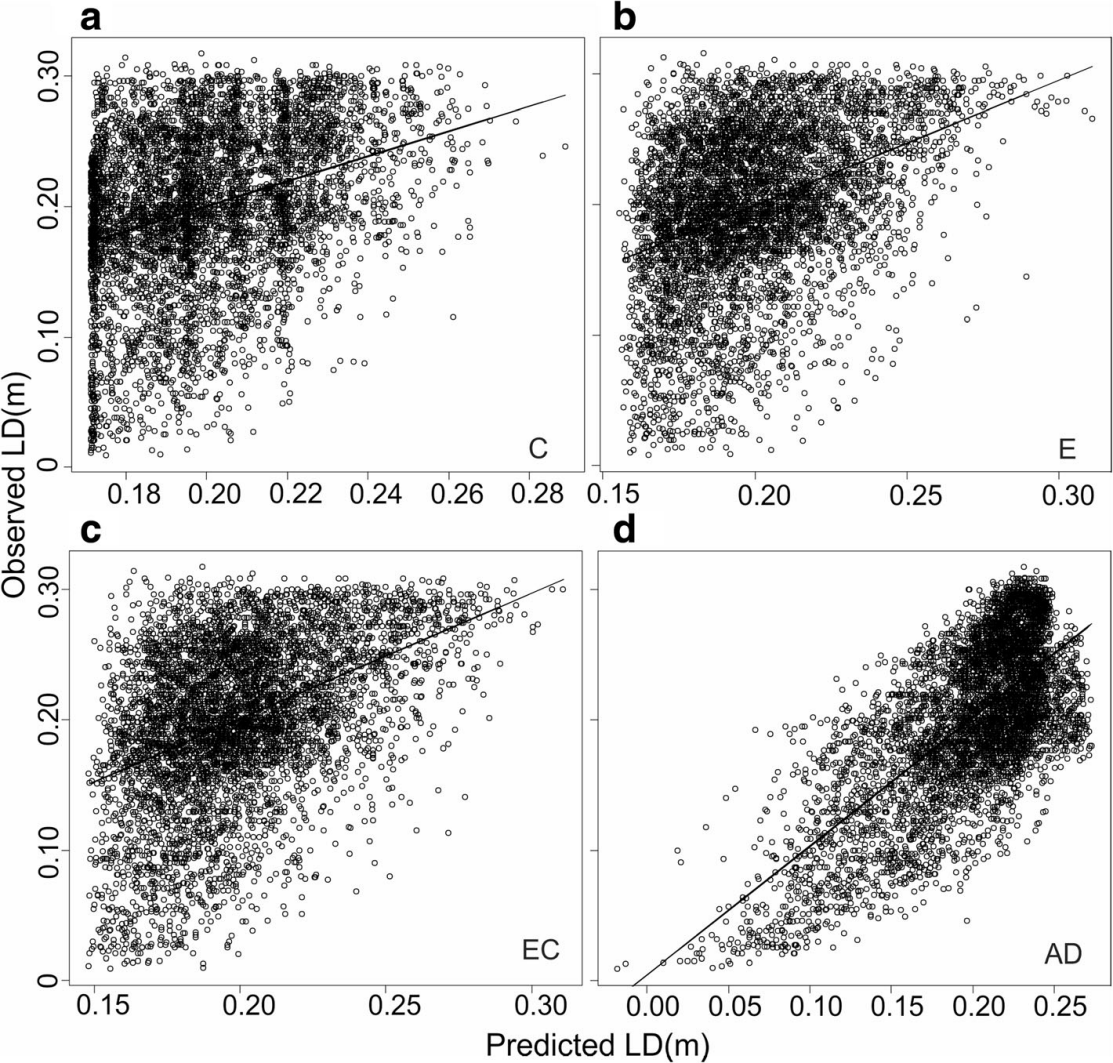
Observed vs predicted linguistic differences between the municipal dialects. Predicted differences between the municipal dialects calculated on the basis of (**a**) cultural differences (corresponds to fractions *b, d, f* and *g* in Fig. 2b), (**b**) environmental differences (corresponds to fractions *a, d, e* and *g* in Fig. 2b), (**c**) environment-cultural differences (corresponds to fractions *a, d, e* and *g* in Fig. 2c), (**d**) administrative and geographical distances (fractions *b, c, d, e, f* and *g* in Fig. 2c). Cultural and environmental features are given in Table 2. Pairing up the 471 municipal dialects resulted in 110,685 linguistic difference values, from which we randomly sampled 5% of the data points for the graphs, leading to *N* = 5535

Cultural differences explained the largest individual fraction of linguistic differences between the dialect groups (Fig. 2a), whereas they explained the smallest fraction of the linguistic differences between the municipal dialects (Fig. 2b). The cultural features included in the final models at both levels described land cover (farmed area and forest land) and house type (chimneyless huts), while in the analysis of the municipal dialects an additional feature related to a subsistence type was also included (slash-and-burn agriculture; Table 2). Thus, features related to land use were left in the models at both levels. To visualise the relationship of linguistic and cultural differences, we correlated the observed linguistic differences with values of linguistic difference predicted by the cultural differences left in the final models (Figs. 3a and 4a; features in the final models are indicated in Table 2). Cultural differences predicted the differences between the dialect groups well (Fig. 3a), but they predicted differences between the municipal dialects well only when the cultural differences were moderate or large (Fig. 4a).

**Table 2:** Environmental and cultural features remaining in the final models

	Dialect groups	Municipal dialects	
	E + C + D	E + C + D	A + D + EC
Cultural	Farmed area^a^ Chimneyless huts^b^	Forest land^a^ Chimneyless huts^b^ Slash-and-burn^c^	Forest land^a^
Environmental	Moraine^a^ Bedrock^a^ River length^d^	Clay^a^ Bedrock^a^ Lake^a^	Clay^a^ Bedrock^a^ Lake^a^

^a^percentage of the total land area ^b^percentage of all residential buildings ^c^per 100 ha of cultivated land ^d^km per total land area

Environmental differences alone explained the second largest fraction of the linguistic differences at both levels: ca. 11 % of the differences between the dialect groups (Fig. 2a) and 6 % of differences between the municipal dialects (Fig. 2b). Environmental features in the final models of both levels described soil type and water systems (Table 2). Large and moderate differences in the environmental conditions predicted linguistic differences well at the dialect group level as well as at the municipal dialect level, while small differences in the environmental conditions were poor predictors of linguistic difference between the municipal dialects (Figs. 3b and 4b).

Environmental and cultural differences together explained over half of the differences between the dialect groups (Fig. 2a). The model fit assessed from the correlation between the predicted and the observed differences between the dialect groups improved when predictions were done with both environmental and cultural features left in the final models (Fig. 3c). In the models of the municipal dialects, environmental and cultural differences explained one fifth of the variation in the linguistic differences (Fig. 2b and c). In the model where environmental and cultural differences were analysed jointly (Fig. 2c), the features remaining in the model described land cover, soil type and water systems (Table 2). The model fit was good with moderate and large environmental and cultural differences, but failed to predict the differences in the municipal dialects with small differences in environmental and culture (Fig. 4c).

**Fig. 5:**
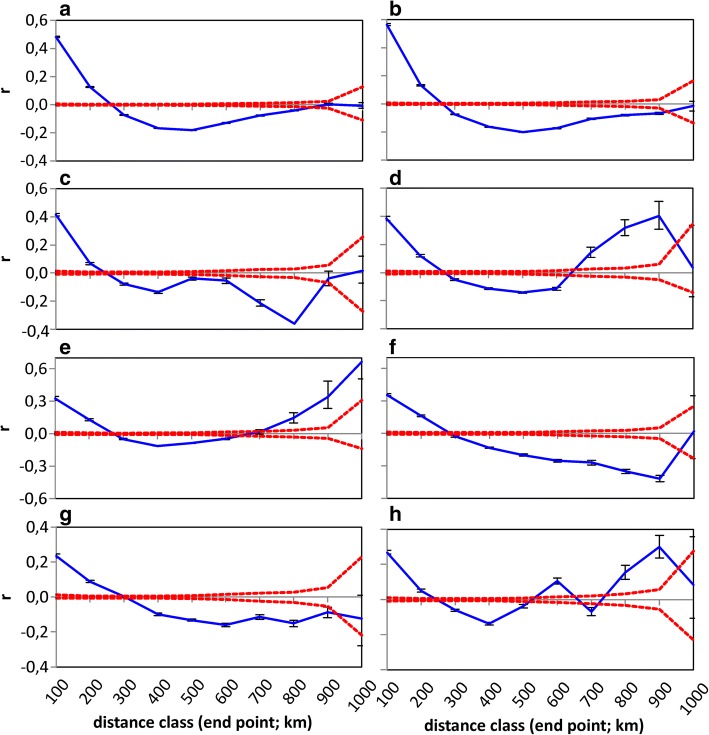
Spatial autocorrelation plots for features remaining in the final model of municipal dialect analysis. Autocorrelation coefficient (*r*; blue line) as a function of geographical distance for (**a**) linguistic differences, (**b**) difference in administrative history, difference in (**c**) percentage of forest land, (**d**) percentage of chimneyless huts, (**e**) slash-and-burn agriculture per 100 ha cultivated land, (**f**) percentage of clay, (**g**) percentage of bedrock and (**h**) percentage of lakes. The 95% confidence intervals calculated with permutation (red lines), and bootstrapped 95% confidence intervals (error bars) are also shown

**Fig. 6:**
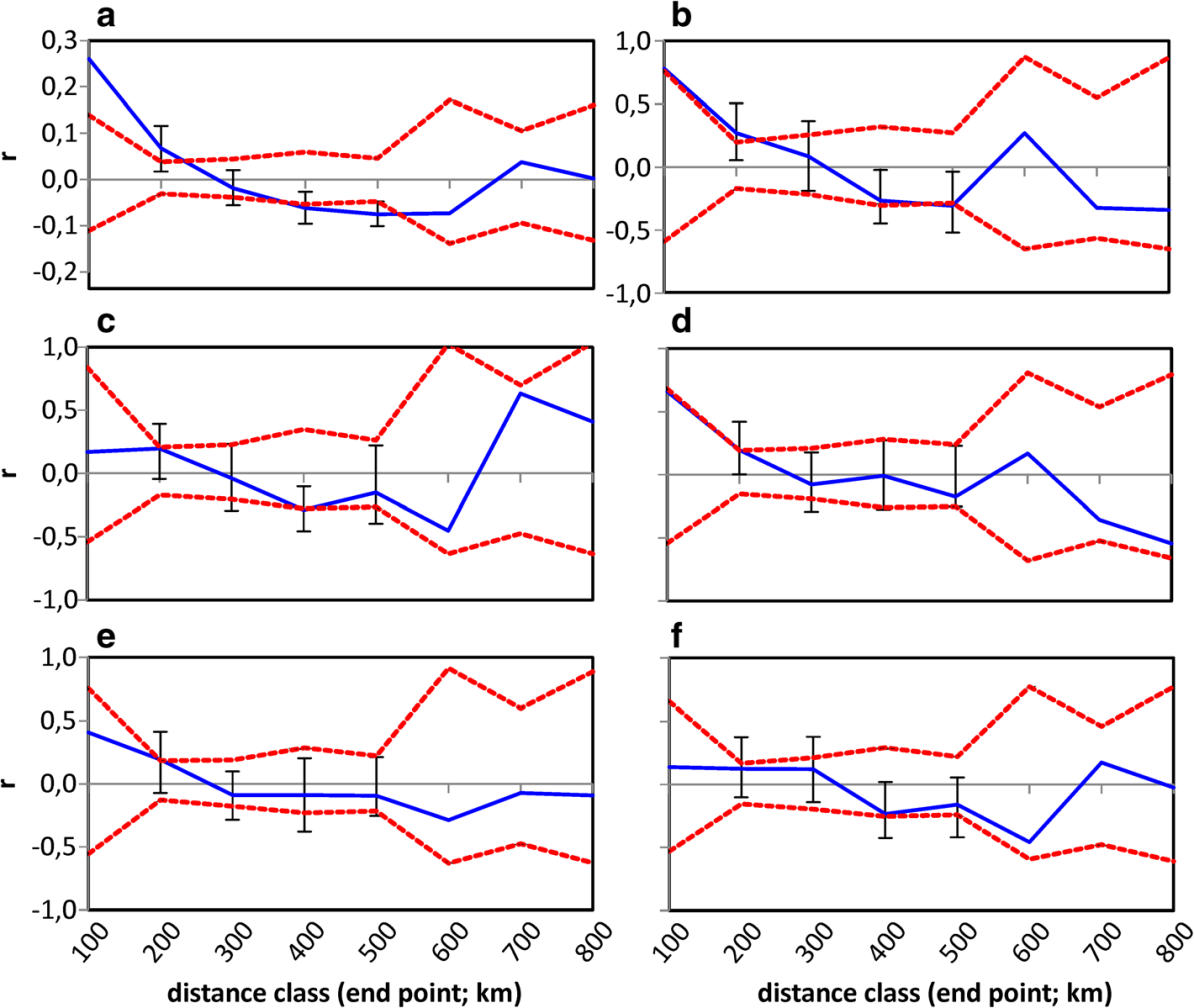
Spatial autocorrelation plots for features remaining in the final model of dialect group analysis. Autocorrelation coefficient (*r*; blue line) as a function of geographical distance for (**a**) linguistic differences, differences in percentage of (**b**) farmed area, (**c**) chimneyless huts, (**d**) moraine, (**e**) bedrock and (**f**) difference in the length of rivers. The 95% confidence intervals calculated with permutation (red lines), and bootstrapped 95% confidence intervals (error bars) are also shown

Geographical distance explained individually only a small part of the differences between the dialect groups (Figs. 2a and 3d). Instead, it explained by far the largest fraction of the linguistic differences between the municipal dialects (Fig. 2b), but the vast majority of this fraction was also explained by administrative distance (Figs. 2c and 4d). The large role of geographical distance at the municipal level is also seen as a highly significant positive spatial autocorrelation of all the dependent and explanatory variables remaining in the final model, especially within the range of 100 km (Fig. 5). At the dialect level, positive spatial autocorrelation is highly significant (*p* = 0.001) within a 100 km range only in linguistic differences between dialect groups (Fig. 6). Administrative distances alone explained a tiny fraction of the variation among the differences of the municipal dialects (Fig. 2c).

## Discussion

We found that environmental differences explained linguistic differences between the dialect groups independently of other explanatory variables. Environmental differences explained even more of the linguistic differences than geographical distances did. In biology, such an observation of IBE suggests genetic differentiation of populations through adaptation to local environment [23, 24, 29], while the pattern of IBE may emerge also for example via assortative mating or phenotypic plasticity without genetic adaptation to the local environment [23].

We consider linguistic IBE to be an indication of a process of adaptation in which spatially varying environmental conditions have played a role. We suggest that what we see in our data is that humans have culturally adapted to different environments [33] and in that process, language has behaved as a neutral marker of cultural history for human populations [30]. In our model, cultural differences alone explained more of the linguistic differences between the dialect groups than environmental and geographical distances. This indicates a connection between neutral and non-neutral cultural features and further reinforces the conclusion that cultural adaptation has had a role in the divergence of Finnish dialect groups. In many cases it may be difficult to decouple linguistic and cultural features and infer the direction of causation. However, as we are connecting language and culture to the physical environment of the language speakers and as humans adapt to their environment with culture and not with language, we propose a hypothesis that cultural differences are the cause and linguistic differences are the effect, and not vice versa.

Cultural adaptation (or “cultural adjustment” or “cultural specialisation”) in this context refers to the accumulation of skills that help people to live and survive in a certain environment, but which are not easily transferable to a different type of environment [50]. It parallels with biological adaptation, where a population is genetically specialised to a certain type of habitat where its individuals have better success than in other, different environments, and where the local individuals outcompete the immigrants coming from a different environment [51–53]. Cultural adaptations within Finland could include the subsistence strategies and farming practices used in different parts of the country depending e.g. on the local climate conditions and soil types [54, 55]. Furthermore, the availability of water systems naturally promotes the development of fishing techniques as part of livelihood. In the north, cattle farming, fishing and hunting were the most important subsistence strategies due to unfavourable conditions for crop farming. In the rest of the country, crop farming techniques have varied from several slash-and-burn techniques to field cultivation, depending on the soil and forest type [54, 55]. For example, the slash-and-burn techniques and crop varieties used in large parts of western Finland were not viable in the east, which is why another variant of slash-and-burn technique and specific varieties of crops more applicable to the eastern conditions were adopted from further east [56].

We propose that differences in cultural adaptations, such as subsistence strategies arising from variation in the environmental conditions, could have acted as nonphysical barriers and limited the contacts between groups. Once at least partial isolation has taken place, the languages of these groups may differentiate in time due to the usual processes of language change (e.g. borrowing, grammaticalisation, sound change and semantic change motivated by e.g. prestige and/or the principle of least effort [57]). This follows the logic presented by Michalopoulos [50] and Gavin et al. [58], who suggested that cultural specialisation may produce distinct cultural spheres, which may in turn lead to group boundary formation, with linguistic diversification as a side product. Finally, it could be said that the language-environment connection found with moderate and large differences in our models is notable, especially when considering that even the largest environmental and cultural differences within Finland are subtle compared to cases where several languages and larger geographical areas are studied [58].

The role of geographical distance in separating the Finnish dialect groups was relatively small. This can be explained at least partly by what is currently known about the arrival and the spread of the Finnish language within Finland. The speakers of the early forms of Finnish arrived in the southern parts of Finland from two directions, from the south across the Gulf of Finland, and from the southeast along the Karelian Isthmus. As a result, early forms of Finnish were spoken in both eastern and western areas about one thousand years ago [59]. The early language varieties spread throughout Finland from these speaker populations. The east-west division remained in the southern and central parts of the country and today it still remains the main linguistic division of Finnish [12]. The northern areas, instead, have had influence from both western and eastern dialects [12, 60]. As a result, the neighbouring eastern and western dialect groups in the different sides of the east-west border are geographically very close, but linguistically very different. In contrast, the northern and southern areas, which are geographically far from each other, share many linguistic features and have comparably small linguistic differences [5].

While pure geographical distance had only a small influence on separating the dialect groups, it explained the largest fraction of the linguistic differences between the municipal dialects. Furthermore, linguistic differences between municipalities showed strong spatial structure over short geographical distances. Similar differences in linguistic IBD across two levels have been detected in Northern Island Melanesia, where linguistic IBD was detected at a local scale but not at a wider scale [61]. On the other hand, linguistic IBD has been found in a global sample of typological features between languages [36]. Thus, in general, the findings regarding the spatial scale at which linguistic IBD has been detected are not consistent.

When we added administrative history to the model of differences between the municipal dialects, the relative importance of pure geographical distance decreased dramatically. Geographical distances and differences in administrative history then jointly explained the majority of the variation in the linguistic differences, while the individual fractions of geographical distance and administrative differences were comparably small. Geographical distance physically limits dispersal between locations while administrative history may form both physical and nonphysical barriers. As the limits of dispersal between municipalities arise now via two mechanisms, they are likely to be more efficient limitations for dispersal than either of these would be alone.

In this study, we have adopted certain elements of the biological microevolutionary approach in order to study and understand the first steps of linguistic divergence. While new dialects, and linguistic diversity in general, may emerge also via contact (e.g. koineization [62]), we focus here only on divergence via isolation. Within this framework, we were able to examine the relative contributions of several factors and separate their individual and joint influences from each other; for example, to what extent do environmental differences contribute to the divergence of dialect groups, and how much of this is due to the geographical distance between locations. In addition, it allowed us to infer the relative contributions of underlying processes from the observed patterns in order to understand the mechanisms of linguistic divergence better. We inferred cultural adaptation from linguistic IBE, but we are not willing to try to pinpoint exactly which environmental or cultural differences would be the most relevant ones in this context, as several environmental and cultural variables are inherently interlinked with each other. Furthermore, to evaluate more thoroughly the inference of adaptive processes from the correlation of linguistic IBE, it would be important to study more specifically the connection between environmental variables and cultural adaptation. This way both neutral (language) and non-neutral (cultural variables under selection) variation would be covered in a comparable way, similar to how the study of both neutral and non-neutral genetic variation has been recommended when detecting the mechanisms behind IBE [29]. We found the role of geographical distance to be rather small in separating dialect groups. However, the relative contribution of geographic distance could be re-evaluated by use of a measure of functional connectivity [63]. It accounts for the time and difficulty of travel (i.e. where it is most cost-effective to move) and takes into consideration for example the promoting influence of waterways and the effects of administrative boundaries on human movement.

## Conclusions

Our results suggest that mere differences in environmental conditions – and adaptive processes related to these – have a larger role in the divergence of the dialects groups than that of geographical distance even within an area of only modest environmental variation. We present here a hypothesis where ecological settings of the speaker populations contribute to the divergence of speaker populations, and put forward a framework with which it is possible to test it. This makes it possible to study the universality of this phenomenon and resolve whether it acts as one of the factors underlying the global patterns of linguistic diversity.

## Methods

### Datasets

The linguistic data were obtained from the Dialect Atlas of Finnish [42] collected by Lauri Kettunen in the 1920s and 1930s. The Atlas contains 213 map pages showing various linguistic features (phonological, morphological and lexical) and their variants in 525 Finnish-speaking municipalities. Within each municipality, Kettunen recorded the linguistic variants of 1–4 informants. Therefore, each municipality has at minimum one variant and at maximum four variants of each linguistic feature. The number of linguistic variants per feature varied between 2 and 15 over the whole study area.

For the present analyses, we excluded the municipalities not within the administrative territory of Finland at the beginning of the twentieth century (Kven, Meänkieli, Ingrian, Karelian), and the islands in the Gulf of Finland, since the explanatory variables data did not cover these areas with uniform quality. Three municipalities in northernmost Lapland, with less than twelve documented features, were also excluded due to an insufficient amount of linguistic data. This reduced the number of municipalities included in this study to 471. The basic study unit of the Atlas is “the linguistic variant in a municipality” resulting in 100,323 potential study units. However, the data was missing from 5.8% of these.

To make the dialect atlas data appropriate for the population genetic analyses we parallelled municipalities and their municipal dialects (*n* = 471) with genomes of biological individuals, linguistic features (*n* = 213) with genetic loci, and the variants of these features (2–15) with alleles. Instead of using only one linguistic variant (allele) per municipality, we analysed the data in a diploid form and included two variants per municipality. A more detailed explanation of the data, its digitisation and its transformation to the diploid form is given in Syrjänen et al. [5]. The digitised atlas is archived in the AVAA-service (http://avaa.tdata.fi/).

We collected data for 22 cultural and 18 environmental explanatory variables from each municipality (Additional file 1: Table S1, Additional file 2). The cultural variables, which also included demographic variables, were related to human populations (including e.g. birth rates and per capita taxes), while the environmental variables represented the physical and natural environment of the municipality, for example temperature and topography. It is, however, important to note the interconnected nature of certain cultural and environmental variables. For example, the coverage of agricultural fields (classified as a cultural variable, since forests are converted into fields by humans) also depends on soil type and thermal conditions (classified as environmental variables). These data were mainly collected from statistical yearbooks going back approximately one century and historical atlases of Finland. However, data on relatively unchanging physical variables, such as soil type, lake coverage and topography were based on modern geographical databases (see Additional file 1: Table S1 for the full list of variables).

The data on administrative history were a compilation of administrative areas and their borders in the territory of Finland from the thirteenth to the nineteenth century. The data included the approximate eastern border after the second Swedish Crusade to Finland around 1250; the division established by the Treaty of Nöteborg between Sweden and the Novgorod Republic in 1323; provincial divisions from the years 1475, 1540, 1635, 1721, 1743, 1747, 1776, 1812 and 1831; bishoprics from the years 1554, 1850 and 1895; and judicial territories from 1634 and 1776 [43, 64, 65] (Additional file 2).

Geographical distances between each pair of municipal dialects were calculated between their respective municipal population centres (Additional file 3). Between the dialect groups, these distances were calculated between the frequency–weighted centroids of the core areas of each group (obtained from the Structure analysis, see below).

### Calculating linguistic differences (dependent variables)

The Dialect Atlas data were turned into distance matrices for the distance-based analyses. The analyses were done separately for the data sets of municipal dialects and of dialect groups. In the analysis of municipal dialects, we used linguistic differences between all 471 municipal dialects as the dependent variable (Additional file 4). Linguistic differences were calculated for each pair of municipal dialects by summing the number of disagreements in dialectal features in that pair and dividing it by the total number of dialectal features. Thus, the calculation was a rough equivalent of Séguy’s distance calculation formula [49].

For the analysis of the dialect groups, the data of municipal dialects were clustered. We did this with a Bayesian clustering technique implemented in Structure (v.2.3.3) [66], a model-based method designed to cluster population genetic data. The suitability of this method of analysis for dialect data has been shown by Syrjänen et al. [5]. Our dataset has less data (471 municipal dialects) than that in Syrjänen et al. ([5]; 525 municipal dialects); for this reason we re-ran the clustering analyses for this study. We clustered the linguistic data into 1–20 clusters (i.e. dialect groups) to estimate the optimal number of dialect groups for the data. We used the admixture model, which allows individuals to originate from more than one population (i.e. in the case of our linguistic data it allows the linguistic variants of a municipal dialect to originate from more than one dialect group). Each municipal dialect was therefore assigned a proportionate membership (a value of Inferred Cluster (IC) ranging from 0 to 1) of each of the dialect groups, allowing the appearance and illustration of transitional dialect areas between the core areas of the dialect groups. We repeated the analysis for each K value ten times to ensure that the results were consistent. The burn-in period was set at 10,000 generations and the number of MCMC repetitions after burn-in at 100,000 generations.

We estimated the optimal number of dialect groups with both the ΔK and the ln Pr (X|K) methods [67]. The results were largely similar to those in Syrjänen et al. ([5]; Fig. 5). Here we considered K = 14 to be the optimal number of dialect groups, as K = 14 got consistently higher maximum likelihood values (6 out of 10 repetitions) than K = 15 did (1 out of 10). Thus, the number of studied units in our analysis of dialect groups was 14. Of the ten repetitions of K = 14 we took the individual run with the highest likelihood to represent the dialect groups. To highlight linguistic differences we used only the core areas of our dialect groups in our analyses (Fig. 1). These were determined by including the municipalities with IC-values > 0.75 in the core areas of the dialect groups while discarding the rest that belonged to the transitional dialect areas.

We used the linguistic differences between the dialect groups as our dependent variable. The differences between the dialect groups were calculated as F_ST_ values (GenAlEx, v.6.41) [68, 69] between each pair of the cores of the dialect groups obtained from the Structure analysis (Fig. 1; Additional file 5). We borrowed the F_ST_ formula from population genetics, where it provides a measure of genetic differentiation between populations [5]. A higher number of differing linguistic variants produced a larger linguistic difference with both distance metrics (Séguy and F_ST_).

### Environmental, cultural, geographical and administrative distances (independent variables)

To compare the differences in environmental and cultural conditions with the linguistic differences between the dialect groups and between the municipal dialects, we transformed all the cultural and environmental variables into Euclidean distance matrices.

For the analyses of municipal dialects, we calculated Euclidean distances between each pair of municipalities for each of the 40 variables (e.g. the difference in the mean temperature (in °C) between each pair of municipalities). For the analyses of the dialect groups, we used data on 33 environmental and cultural variables (see Additional file 1: Table S1, Additional file 5). We first calculated averages of these variables for each core dialect area (e.g. the average temperature of Häme). Instead of averages, we calculated sums of core municipalities for the total population number and area of the municipality (Additional file 1: Table S1). Subsequently, these values were transformed into Euclidean distances between each pair of dialect groups for each of the 33 variables.

Geographical distances between the municipal dialects were calculated as planar straight-line distances between municipal population centres. These were determined manually as the location of the largest or main settlement in each municipality at the turn of the nineteenth and twentieth century [70].

For dialect groups, geographical distances were calculated between the dialect group frequency (IC) –weighted centroids of the core areas of the dialect groups (based on values of IC > 0.75) (Additional file 5). These centroids were determined as a weighted arithmetic means from the coordinates of the municipal population centres within the dialect group and the IC value of the dominant dialect group in these municipalities. Thus, instead of having the geographical centroid of the dialect group, the centroid was shifted towards the municipalities with the highest IC values. A planar distance matrix was calculated between these weighted centroids.

The administrative data were turned into a Jaccard distance matrix to represent differences in administrative histories between each pair of municipalities. The variables were coded in a binary form where a municipality either belonged to a particular administrative area, e.g. the province of Savonlinna in year 1475 (=1) or it did not (=0) (Additional file 2). This binary coding was turned into Jaccard index values by taking a pair of municipalities, summing the number of times these municipalities had belonged to different administrative areas (over all the 16 administrative divisions used here), and dividing it with the sum of the administrative areas to which either one or both of the municipalities had belonged. As a result, municipalities with different administrative histories received large Jaccard index values (cf. [71]). All distance calculations (except the F_ST_) were performed with R [72].

Autocorrelation coefficients were calculated for different distance classes (100 km each) with GenAlEx 6.5 [68, 69]. The upper and lower bounds of 95% confidence intervals were obtained with 999 random permutations, and the 95% error bars for each distance class with 999 bootstrap trials.

### Model building

We used multiple regression on distance matrices (MRM; [46, 47]) to analyse whether linguistic differences were explained by geographical distances, differences in environmental and cultural conditions or differences in administrative history. We first transformed geographical distance matrices into log_10_-values for a better fit of the MRM model. We then excluded the cultural and environmental features that did not correlate with linguistic differences or correlated only due to geographical distance. This was done by conducting one-tailed partial Mantel tests [73] between the linguistic differences and each of the explanatory variables with the effect of the logarithm of the geographical distance taken into account. The tests were conducted with 1000 permutations each with the ‘ecodist package’ [74] for R [72] at both the levels of municipal dialects and dialect groups.

Finally, we determined the multicollinearity of all the remaining cultural and environmental variables. We studied the multicollinearity of the variables at the municipality level with Spearman correlations. In cases of high correlations (Spearman correlation coefficient > 0.7 [75], see Additional file 6: Table S2), variables which had the highest correlations with several other variables were excluded, and only one of the inter-correlated factors was left in the model to represent these variables. In total five environmental and five cultural variables were left for the MRM analysis at the level of municipal dialects (Additional file 1: Table S1). A similar selection for uncorrelated explanatory variables was conducted for dialect groups but with a higher limit for correlations (correlation coefficient > 0.9; see Additional file 7: Table S3). Altogether five environmental and three cultural variables were left for the model selection at the dialect group level (Additional file 1: Table S1).

With these procedures, we selected for MRM analyses those environmental and cultural features which correlated with linguistic differences but were not too correlated with each other. Prior to analysing the data with MRM, the features were standardised to make the coefficients mutually comparable.

### MRM analyses and variation partitioning

To determine the relative impacts of the differences in the explanatory variables on linguistic differences, we ran three sets of MRM analyses: one for differences between the dialect groups and two for differences between the municipal dialects.

In the analyses of the dialect groups, we explained the differences between dialect groups with environmental and cultural differences and with geographical distance. Here, MRM was first used in model selection to find the environmental and cultural features that best explained the linguistic differences and that would therefore be included in the final environmental model and the final cultural model. Model selection was done separately for environmental and cultural features with a backward elimination procedure [46], starting with five environmental and three cultural features (Additional file 1: Table S1). We removed the feature with the lowest coefficient in each round until the R^2^ value dropped dramatically, resulting with a positive regression coefficient for all the features remaining in the models. Hereby, the final environmental and the final cultural model had the largest explanatory power with the least number of features. From this analysis, we obtained the R^2^ values for the final individual models of environment (including two features) (E) and culture (three features) (C). We also ran the MRM to obtain R^2^ for the logarithm of the geographical distance. We then combined the features left in the final individual models to calculate the explanatory powers of the combined models: “environment and culture model” (EC; five features), “environment and geographical distance model” (ED; three features), “culture and geographical distance model” (CD; four features) and “environment, culture, and geographical distance model” (ECD; six features).

In the first set of analyses of the municipal dialects, we explained linguistic differences between the municipal dialects with environmental and cultural differences and with geographical distance. Here, to obtain the final environmental and cultural models we again began with model selection, starting with five environmental and five cultural features for the municipalities (Additional file 1: Table S1). The final environmental and cultural models included three features each and their R^2^ values were calculated. As above, we used MRM to get R^2^ values for the logarithm of the geographical distance individually, and for the combined models (EC, six features; ED, four features; CD, four features; ECD; seven features).

In the second set of analyses of the municipal dialects, we explained linguistic differences between the municipal dialects by administrative differences (A), geographical distances (D) and a joined environment-culture model. Here, model selection was done jointly for environmental and cultural features with a backward elimination procedure starting with 10 features in total. Four features (three environmental and one cultural) were left in the final environment-culture (EC) model (Additional file 1: Table S1). Again, R^2^ values were calculated for each of the individual models (A, D, EC) and the combined ones (AD; two features, AEC; five features, DEC; five features, ADEC; six features). MRM calculations were performed with the ‘ecodist package’ of R [74], with 1000 permutations each.

MRM models show the relative importance of different explanatory factors. However, to resolve how much different factors explain of the linguistic variation individually (e.g. pure environmental effect) and how much is explained jointly by two or three factors (e.g. environment explains together with geographical distance), we performed variation partitioning [47, 76]. These fractions were calculated from the R^2^ values obtained from MRM analyses (for the methodology, see Macía et al. [77] and Heikkinen et al. [78]).

## Additional files

Additional file 1: **Table S1.** Explanatory variables and their usage in the analyses. * Abbreviations used in Tables S2 and S3. Variables without abbreviations were eliminated in the partial Mantel analyses. † Variables which were left after the partial Mantel test and correlation observations and were inserted to MRM analyses. ‡ Variables left in the final models. § Variables from which averages and sums were calculated for the municipality data. ¶ Sums of per municipality (instead of average) values were calculated for these variables. (DOCX 19 kb)

Additional file 2: Environmental, cultural and administrative data used in the analyses of municipal dialects. Raw data of 40 environmental and cultural variables (sheet 1), and binary data on administrative history (sheet 2) used in the analyses of municipal dialects in 471 municipalities. (XLS 805 kb)

Additional file 3: Geographical distances between municipalities. Planar straight-line distances between municipal population centres (in metres). (CSV 1490 kb)

Additional file 4: Linguistic distances between municipalities. Rough equivalents of Séguy’s dialect distance metric, i.e. a percentage of disagreeing linguistic features between pairs of municipal dialects. (CSV 1277 kb)

Additional file 5: Datasets of the dialect group analyses. Linguistic F_ST_ distances (sheet 1), geographical distances (sheet 2) and raw data of 33 environmental and cultural variables (sheet 3) used in the analyses of the dialect groups. (XLS 52 kb)

Additional file 6: **Table S2.** Spearman correlations of municipality raw data for variables remaining after partial Mantel test. (DOCX 16 kb)

Additional file 7: **Table S3.** Pearson and Spearman correlations of the 14 dialect groups for variables remaining after partial Mantel test. *Spearman correlations. (DOCX 13 kb)

## Abbreviations

IBA: Isolation by administrative history
IBC: Isolation by culture
IBD: Isolation by distance
IBE: Isolation by environment
MRM: Multiple regression on distance matrices
