# Supplementary material for: Evolution within a language: environmental differences contribute to divergence of dialect groups
Source: BMC Evol Biol. 2018 Sep 3;18:132. doi: 10.1186/s12862-018-1238-6 (PMC6122686; doi:10.1186/s12862-018-1238-6)
Supplement: Supplementary file 1 — Table S1. Explanatory variables and their usage in the analyses. * Abbreviations used in Tables S2 and S3. Variables without abbreviations were eliminated in the partial Mantel analyses. † Variables which were left after the partial Mantel test and correlation observations and were inserted to MRM analyses. ‡ Variables left in the final models. § Variables from which averages and sums were calculated for the municipality data. ¶ Sums of per municipality (instead of average) values were calculated for these variables. (DOCX 19 kb) [file 12862_2018_1238_MOESM1_ESM.docx]

|  |  | Municipality | | | Dialect area K14 | | |
| --- | --- | --- | --- | --- | --- | --- | --- |
| **Environmental** | Abbrev.* | To MRM† | Final AECD‡ | Final  ECD‡ | Aver/ sums§ | To MRM† | Final ECD‡ |
| Mean annual temperature (ºC) [1] |  |  |  |  | x |  |  |
| Annual precipitation (mm) [1] |  |  |  |  | x |  |  |
| Number of rainy days per year (> 1 mm) [1] |  |  |  |  | x |  |  |
| Depth of snow cover (cm) [1] | snowdep |  |  |  | x |  |  |
| Duration of snow cover (days) (years) [1] | snowdays |  |  |  | x |  |  |
| The current annual increment per hectare of forest land (m^3^) [1] |  |  |  |  | x |  |  |
| Growing stock on an average per hectare of forest (m^3^) [1] |  |  |  |  | x |  |  |
| The average quality of forest lands. The annual productive capacity when the forests are not thinned (m^3^/ha) [1] |  |  |  |  | x |  |  |
| Land area of the municipality (ha) [2-4] |  |  |  |  | x¶ |  |  |
| Mean height of the municipality (meters above sea level) [4, 5] |  |  |  |  | x |  |  |
| Lakes (% of total land area of a municipality) [4, 6] | lake | x | x | x | x | x |  |
| Rivers (total river lengths per total land area of a municipality) [4] | river | x |  |  | x | x | x |
| Moraine (% of total land area of a municipality) [1, 7] | morai | x |  |  | x | x | x |
| Clay (% of total land area of a municipality) [1, 7] | clay | x | x | x | x | x |  |
| Gravel or sand (% of total land area of a municipality) [1, 7] |  |  |  |  | x |  |  |
| Bedrock (% of total land area of a municipality) [1, 7] | rock | x | x | x | x | x | x |
| Peat (% of total land area of a municipality) [1, 7] |  |  |  |  | x |  |  |
| Heat summation (ºC) [8] |  |  |  |  | x |  |  |
| **Cultural** |  | | | | | | |
| Forest land (% of total land area of a municipality) [2] | forest | x | x | x | x | x |  |
| Fields (% of total land area of a municipality) [2] | field |  |  |  | x |  |  |
| Birth rate (annual mean for 1000 inhabitants) [1] |  |  |  |  | x |  |  |
| Death rate (annual mean for 1000 inhabitants) [1] |  |  |  |  | x |  |  |
| Infant mortality: infants died during their first year of one´s life (annual mean for 1000 born child) [1] |  |  |  |  | x |  |  |
| Meadows (% of total land area of a municipality) [2] |  |  |  |  | x |  |  |
| Forests, wasteland etc. (% of total land area of a municipality) [2] | forestwas |  |  |  | x |  |  |
| Slash and burn area growing grain per 100 ha of cultivated land [2] | slashburn | x |  | x | x |  |  |
| Average of inhabitants per residential building [9] |  |  |  |  | x |  |  |
| Chimneyless peasant huts (% of all residential buildings) [9] | nochim | x |  | x | x | x | x |
| Immigration per 1000 inhabitants [9] | immi |  |  |  |  |  |  |
| Emigration per 1000 inhabitants [9] | emig |  |  |  |  |  |  |
| Number of Finnish speaking inhabitants [10] | finn |  |  |  |  |  |  |
| Number of Swedish speaking inhabitants [10] |  |  |  |  |  |  |  |
| Total population number [10] | totpop | x |  |  | x¶ |  |  |
| Population density (total population/area) [2, 10] |  |  |  |  | x |  |  |
| Farmed area (% of total land area of a municipality [2] | farme |  |  |  | x | x | x |
| Population increase (immigration + birth rate) [9] | popincr |  |  |  |  |  |  |
| Population decrease (emigration + death rate) [9] | popdecr | x |  |  |  |  |  |
| Population change (increase - decrease) [9] |  |  |  |  |  |  |  |
| Income per capita [11-13] |  |  |  |  | x |  |  |
| Taxes per capita [11-13] |  |  |  |  | x |  |  |

**Additional file 1. Table 1**

**References**

1. Suomen Maantieteellinen Seura. Suomen Kartasto 1925 [Atlas of Finland 1925]. Helsinki: Otava; 1929.

2. Suomen Virallinen Tilasto. 3, Maatalous. 9. Maataloustiedustelu Suomessa vuonna 1910. Helsinki: Maataloushallitus; 1916.

3. Central Statistical Office of Finland. Population by Industry: Population by Industry and Commune in 1880–1975. Helsinki: Tilastokeskus; 1979.

4. National Land Survey Finland: The Topographic database. 2012. http://www.maanmittauslaitos.fi/en/digituotteet/topographic-database. Accessed 7 July 2012.

5. Jarvis A, Reuter HI, Nelson A, Guevara E: Hole-filled SRTM for the globe Version 4. 2008. http://srtm.csi.cgiar.org. Accessed 19 Oct 2012.

6. Geofabrik & OpenStreetMap contributors: OpenStreetMap Data Extracts. 2013. http://download.geofabrik.de/. Accessed 17 Dec 2013.

7. Geological Survey of Finland: Soil map 1:20 000 WFS service. 2012. http://www.gtk.fi/tietopalvelut/karttapalvelut/rajapintapalvelut.html. Accessed 11 Dec 2013.

8. Alalammi P, Winqvist S, Ahonen K. Atlas of Finland, 131, Climate. Helsinki: National Board of Survey; 1988.

9. Official Statistics of Finland. Central Statistical Office of Finland; 1889.

10. Tilastollinen toimisto. Suomenmaan Tilastollinen vuosikirja 1882 [Statistical Yearbook of Finland 1882]. Helsinki: Suomalaisen Kirjallisuuden Seura; 1882.

11. Tilastollinen päätoimisto. Suomen Virallinen Tilasto IV C, Kunnallinen Verotus Vuoden 1924 Tuloista [Official Statistics of Finland. IV C, Municipal Taxation of Year 1924 Income]. Helsinki; 1928.

12. Tilastollinen päätoimisto. Suomen Virallinen Tilasto IV C, Kunnallinen Verotus Vuoden 1927 Tuloista [Official Statistics of Finland. IV C, Municipal Taxation of Year 1927 Income]. Helsinki; 1931.

13. Tilastollinen päätoimisto. Suomen Virallinen Tilasto IV C, Kunnallinen Verotus Vuoden 1932 Tuloista [Official Statistics of Finland. IV C, Municipal Taxation of Year 1932 Income]. Helsinki; 1935.
