# Supplementary material for: Evolution within a language: environmental differences contribute to divergence of dialect groups
Source: BMC Evol Biol. 2018 Sep 3;18:132. doi: 10.1186/s12862-018-1238-6 (PMC6122686; doi:10.1186/s12862-018-1238-6)
Supplement: Supplementary file 6 — Table S2. Spearman correlations of municipality raw data for variables remaining after partial Mantel test. (DOCX 16 kb) [file 12862_2018_1238_MOESM6_ESM.docx]

|  | rock | morai | clay | snow  dep | snow  days | lake | river | finn | tot  pop | forest | field | farme | forest  was | slash  burn | no  chim | immi | pop  incr | emig | pop  decr |
| --- | --- | --- | --- | --- | --- | --- | --- | --- | --- | --- | --- | --- | --- | --- | --- | --- | --- | --- | --- |
| rock | 1 |  |  |  |  |  |  |  |  |  |  |  |  |  |  |  |  |  |  |
| morai | -0.48 | 1 |  |  |  |  |  |  |  |  |  |  |  |  |  |  |  |  |  |
| clay | 0.41 | -0.65 | 1 |  |  |  |  |  |  |  |  |  |  |  |  |  |  |  |  |
| snow  dep | -0.41 | 0.50 | -0.75 | 1 |  |  |  |  |  |  |  |  |  |  |  |  |  |  |  |
| snow  days | -0.50 | 0.58 | -0.78 | 0.93 | 1 |  |  |  |  |  |  |  |  |  |  |  |  |  |  |
| lake | -0.03 | 0.23 | -0.44 | 0.36 | 0.34 | 1 |  |  |  |  |  |  |  |  |  |  |  |  |  |
| river | 0.10 | -0.00 | 0.33 | -0.31 | -0.32 | -0.47 | 1 |  |  |  |  |  |  |  |  |  |  |  |  |
| finn | -0.04 | 0.12 | -0.10 | 0.18 | 0.16 | 0.20 | -0.13 | 1 |  |  |  |  |  |  |  |  |  |  |  |
| tot  pop | -0.02 | 0.13 | -0.06 | 0.14 | 0.12 | 0.18 | -0.10 | 0.95 | 1 |  |  |  |  |  |  |  |  |  |  |
| forest | -0.21 | 0.49 | -0.67 | 0.65 | 0.62 | 0.52 | -0.41 | 0.20 | 0.14 | 1 |  |  |  |  |  |  |  |  |  |
| field | 0.45 | -0.64 | 0.86 | -0.84 | -0.88 | -0.31 | 0.24 | -0.12 | -0.08 | -0.66 | 1 |  |  |  |  |  |  |  |  |
| farme | 0.42 | -0.61 | 0.84 | -0.78 | -0.82 | -0.27 | 0.23 | -0.06 | -0.01 | -0.61 | 0.94 | 1 |  |  |  |  |  |  |  |
| forest  was | -0.32 | 0.59 | -0.79 | 0.72 | 0.70 | 0.34 | -0.23 | 0.09 | 0.04 | 0.66 | -0.86 | -0.93 | 1 |  |  |  |  |  |  |
| slash  burn | -0.10 | 0.23 | -0.36 | 0.38 | 0.32 | 0.43 | -0.29 | 0.32 | 0.29 | 0.46 | -0.32 | -0.26 | 0.30 | 1 |  |  |  |  |  |
| no  chim | -0.29 | 0.34 | -0.50 | 0.58 | 0.57 | 0.47 | -0.36 | 0.26 | 0.21 | 0.50 | -0.49 | -0.43 | 0.41 | 0.52 | 1 |  |  |  |  |
| immi | 0.23 | -0.35 | 0.45 | -0.48 | -0.45 | -0.16 | 0.14 | -0.36 | -0.34 | -0.36 | 0.50 | 0.47 | -0.48 | -0.23 | -0.36 | 1 |  |  |  |
| pop  incr | 0.11 | -0.17 | 0.34 | -0.36 | -0.32 | -0.19 | 0.19 | -0.32 | -0.31 | -0.26 | 0.34 | 0.32 | -0.29 | -0.28 | -0.37 | 0.84 | 1 |  |  |
| emig | 0.25 | -0.32 | 0.46 | -0.52 | -0.48 | -0.13 | 0.19 | -0.37 | -0.34 | -0.36 | 0.52 | 0.50 | -0.49 | -0.23 | -0.33 | 0.85 | 0.70 | 1 |  |
| pop  decr | 0.16 | -0.28 | 0.41 | -0.41 | -0.38 | -0.17 | 0.15 | -0.32 | -0.29 | -0.34 | 0.44 | 0.44 | -0.45 | -0.21 | -0.29 | 0.83 | 0.79 | 0.90 | 1 |

**Additional file 6. Table 2**
