# Supplementary material for: Evolution within a language: environmental differences contribute to divergence of dialect groups
Source: BMC Evol Biol. 2018 Sep 3;18:132. doi: 10.1186/s12862-018-1238-6 (PMC6122686; doi:10.1186/s12862-018-1238-6)
Supplement: Supplementary file 7 — Table S3. Pearson and Spearman correlations of the 14 dialect groups for variables remaining after partial Mantel test. *Spearman correlations. (DOCX 13 kb) [file 12862_2018_1238_MOESM7_ESM.docx]

|  | rock | morai | clay | snow  dep | forest | field | lake | river | farme | no  chim |
| --- | --- | --- | --- | --- | --- | --- | --- | --- | --- | --- |
| rock | 1* |  |  |  |  |  |  |  |  |  |
| morai | -0.64* | 1 |  |  |  |  |  |  |  |  |
| clay | 0.45* | -0.77* | 1* |  |  |  |  |  |  |  |
| snowdep | -0.56* | 0.77 | -0.77 | 1 |  |  |  |  |  |  |
| forest | -0.38* | 0.78 | -0.84 | 0.71 | 1 |  |  |  |  |  |
| field | 0.62* | -0.87* | 0.90* | -0.90* | -0.74* | 1* |  |  |  |  |
| lake | 0.03* | 0.30 | -0.47 | 0.22 | 0.69 | -0.30 | 1 |  |  |  |
| river | 0.16* | -0.34 | 0.55 | -0.54 | -0.74 | 0.45 | -0.68 | 1 |  |  |
| farme | 0.67* | -0.82 | 0.87 | -0.91 | -0.79 | 0.96 | -0.34 | 0.58 | 1 |  |
| nochim | -0.32* | 0.42* | -0.51* | 0.56* | 0.55* | -0.55* | 0.75* | -0.73* | -0.48* | 1* |

**Additional file 7. Table 3**
